# To the Editors of the Medical and Physical Journal

**Published:** 1799-07

**Authors:** W. Woodville

**Affiliations:** Ely-Place


### Medical and Physical Journal.

VOL. I.] JULY, 1799. [NO. V.

### *To the Editors of the Medical and Physical Journal.*

GENTLEMEN,

IT was my intention to have given you a detail of ſeveral particulars reſpecting the cow-pox; but various occurences have prevented me, at preſent, from ſaying more on the ſubject, than what follows:

In my "Reports of Inoculation for the Cow-pox," publiſhed laſt month, it appears that more than one half of the patients had puſtules; I have however obſerved, that the reſult would probably have been more favourable, if the matter uſed for communicating the infection, had been taken from thoſe only, in whom the diſeaſe proved to be very mild.

My ſubſequent experience has now enabled me to ſay, that this opinion has been confirmed; or that the diſeaſe in its progreſs from patient to patient, has actually become much milder. For out of 310 caſes of cow-pox, which have been ſince under my care, only 39 had puſtules that ſuppurated; viz. out of the firſt 100, 19 had puſtules, out of the ſecond 13, and out of the laſt 110, only 7 had puſtules. This information I deem of conſiderable importance, as it leads to a concluſion widely different from that publiſhed in the "Reports."

ELY-PLACE,
*June* 13, 1799.

W. WOODVILLE.

