# Errata in Vol. I

**Published:** 1799-08

**Authors:** 


No. V. p. 418, l. 28, for " professional," read, *professorial* chair."

No. V. p. 419. l. 1, for " 1699," read " 1669."

No. V. p 419, l. 8, for " ingenious," read, " *ingenuous*."

No. V. p. 419, l. 36, near the bottom, in the last line of the Latin quotation, from Thruston, read ' quamque interim aliorum scripta de eadem re edita, vel merita sunt vel ambierunt.'"

